# ‘rtry’: An R package to support plant trait data preprocessing

**DOI:** 10.1002/ece3.11292

**Published:** 2024-05-08

**Authors:** Olee Hoi Ying Lam, Jens Kattge, Susanne Tautenhahn, Gerhard Boenisch, Kyle R. Kovach, Philip A. Townsend

**Affiliations:** ^1^ Department of Forest and Wildlife Ecology University of Wisconsin‐Madison, Russell Laboratories Madison Wisconsin USA; ^2^ Max Planck Institute for Biogeochemistry Jena Germany; ^3^ German Centre for Integrative Biodiversity Research (iDiv) Halle‐Jena‐Leipzig Leipzig Germany

**Keywords:** biodiversity, data cleaning, data preprocessing, plant trait, R package, TRY database

## Abstract

Plant trait data are used to quantify how plants respond to environmental factors and can act as indicators of ecosystem function. Measured trait values are influenced by genetics, trade‐offs, competition, environmental conditions, and phenology. These interacting effects on traits are poorly characterized across taxa, and for many traits, measurement protocols are not standardized. As a result, ancillary information about growth and measurement conditions can be highly variable, requiring a flexible data structure. In 2007, the TRY initiative was founded as an integrated database of plant trait data, including ancillary attributes relevant to understanding and interpreting the trait values. The TRY database now integrates around 700 original and collective datasets and has become a central resource of plant trait data. These data are provided in a generic long‐table format, where a unique identifier links different trait records and ancillary data measured on the same entity. Due to the high number of trait records, plant taxa, and types of traits and ancillary data released from the TRY database, data preprocessing is necessary but not straightforward. Here, we present the ‘rtry’ R package, specifically designed to support plant trait data exploration and filtering. By integrating a subset of existing R functions essential for preprocessing, ‘rtry’ avoids the need for users to navigate the extensive R ecosystem and provides the functions under a consistent syntax. ‘rtry’ is therefore easy to use even for beginners in R. Notably, ‘rtry’ does not support data retrieval or analysis; rather, it focuses on the preprocessing tasks to optimize data quality. While ‘rtry’ primarily targets TRY data, its utility extends to data from other sources, such as the National Ecological Observatory Network (NEON). The ‘rtry’ package is available on the Comprehensive R Archive Network (CRAN; https://cran.r‐project.org/package=rtry) and the GitHub Wiki (https://github.com/MPI‐BGC‐Functional‐Biogeography/rtry/wiki) along with comprehensive documentation and vignettes describing detailed data preprocessing workflows.

## Graphical abstract

‘rtry’ is an open‐source package that simplifies the plant trait data preprocessing workflow. The package was developed as part of the TRY R project to preprocess trait data received via the TRY database and was designed to be applicable to trait datasets from other sources.

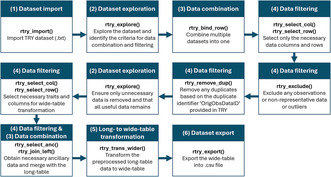

## 1 INTRODUCTION

Traits are characterized as quantities of entities (Entity‐Quality Model; Garnier et al., 2017; Mungall et al., 2010), and plant traits are defined as the morphological, anatomical, physiological, biochemical, and phenological characteristics of plants measurable at the individual plant level (Violle et al., 2007). Traits reflect the outcome of evolutionary, genetic, and community assembly processes responding to abiotic and biotic environmental constraints and determine how individuals perform and respond to environmental factors. Traits thus provide a link from species richness to functional diversity, which influences ecosystem properties and how they affect human beings. To prevent the loss of biodiversity and degradation of ecosystems, studies are increasingly focusing on the collection and analysis of plant traits, which, for example, have been selected as key observations in the context of the US National Science Foundation's National Ecological Observatory Network (NSF's NEON; https://www.neonscience.org) and the Australian land ecosystem observatory (Terrestrial Ecosystem Research Network; https://www.tern.org.au). Due to improved availability, plant traits now extend the range of earth observations to the level of individual organisms, providing a link from biodiversity to ecosystem function and modeling in the context of rapid global changes (Kattge et al., 2020).

### 1.1 A global database of plant traits—TRY

**FIGURE 1:**
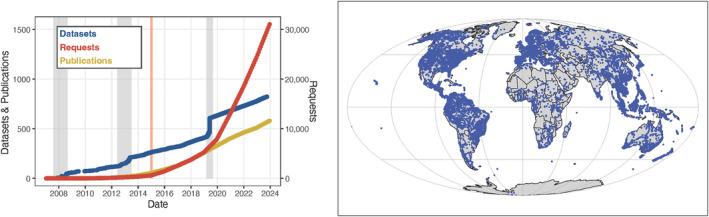
(Left) Cumulative numbers of datasets and publications (left axis), and data requests (right axis); Gray vertical bars indicate the calls for data contribution, while the orange bar indicates the date of opening TRY to the public. (Right) Geographic coverage of measurement sites (blue points) in TRY version 6 in the Mollweide projection.

In 2007, the TRY initiative (https://www.try‐db.org) was launched, aiming at developing a global database of plant traits to support biodiversity research, functional biogeography, and modeling of vegetation dynamics. The TRY database initiative received strong support from the ecological community, who contributed many original and collective datasets and has led to multiple updates (Kattge, Díaz, et al., 2011). The current version of the TRY database (version 6), released in October 2022, is based on 696 datasets and contains 15.4 million trait records, accompanied by 43 million ancillary data records, for 2661 traits and 305,000 plant taxa, mostly at the species level. About 6.7 million trait records are georeferenced from about 48,000 measurement sites worldwide (Figure 1). In 2015, some TRY datasets became public, and since 2019 the data are open access under a Creative Commons (CC)‐BY license by default (Kattge et al., 2020). As of today, the TRY initiative has served more than 30,000 data requests (Figure 1), releasing over 4.5 billion trait records in combination with 40 billion ancillary data records. The TRY database has thus become a central resource for the ecological community, allowing users from around the globe to retrieve plant trait data based on selected traits and species or request individual datasets via the data portal on the TRY website. Step‐by‐step instructions on how to register and request data from the TRY database can be found on the GitHub Wiki of ‘rtry’: https://github.com/MPI‐BGC‐Functional‐Biogeography/rtry/wiki/The‐TRY‐database#request_rtry_data.

Through the data request process, users can navigate the intellectual property guidelines of the database, review the description of the requested traits and species, and ascertain the number of trait measurements before sending out the request. Once the request is approved, users have the option to retrieve the dataset from the portal whenever necessary. The data release notes provided with each data request (https://www.try‐db.org/TryWeb/TRY_Data_Release_Notes.pdf) offer information on the generalities, data structure, column headers (Table 1) of the requested dataset, and the identifiers for some of the widely used ancillary data (‘DataID’). Additionally, users can access descriptions and corresponding identifiers of traits (‘TraitName’ and ‘TraitID’) and species (‘AccSpeciesName’ and ‘AccSpeciesID’) on the TRY data explorer (https://www.try‐db.org/de/de.php). This information, particularly the identifiers, is invaluable for the data preprocessing tasks.

**TABLE 1:** Column headers and descriptions for TRY version 6 released data.^a^

	Column	Description
1.	‘LastName’	Surname of data contributor
2.	‘FirstName’	First name of data contributor
3.	‘DatasetID’	Unique identifier of contributed dataset
4.	‘Dataset’	Name of contributed dataset
5.	‘SpeciesName’	Original name of species
6.	‘AccSpeciesID’	Unique identifier of consolidated species name
7.	‘AccSpeciesName’	Consolidated species name
8.	‘ObservationID’	Unique identifier for each observation in TRY
9.	‘ObsDataID’	Unique identifier for each row in the TRY data table, either trait record or ancillary data
10.	‘TraitID’	Unique identifier for traits (only if the record is a trait)
11.	‘TraitName’	Name of trait (only if the record is a trait)
12.	‘DataID’	Unique identifier for each ‘DataName’ (either sub‐trait or ancillary data)
13.	‘DataName’	Name of sub‐trait or ancillary data
14.	‘OriglName’	Original name of sub‐trait or ancillary data
15.	‘OrigValueStr’	Original value of trait or ancillary data
16.	‘OrigUnitStr’	Original unit of trait or ancillary data
17.	‘ValueKindName’	Value kind (single measurement, mean, median, etc.)
18.	‘OrigUncertaintyStr’	Original uncertainty
19.	‘UncertaintyName’	Kind of uncertainty (standard deviation, standard error, etc.)
20.	‘Replicates’	Number of replicates
21.	‘StdValue’	Standardized trait value: available for frequent continuous traits
22.	‘UnitName’	Standard unit: available for frequent continuous traits
23.	‘RelUncertaintyPercent’	Relative uncertainty in %
24.	‘OrigObsDataID’	Unique identifier for duplicate trait records
25.	‘ErrorRisk’	Indication for outlier trait values: distance to mean in standard deviations
26.	‘Reference’	Reference to be cited if trait record is used in analysis
27.	‘Comment’	Explanation for the ‘OriglName’ in the contributed dataset

^a^ Note that sometimes R may show a column 28, which should be empty. This column is an artifact due to the different interpretations of column separator by MySQL and R.

### 1.2 Structure of datasets released from TRY

Plant traits provide essential information about plant growth strategies and adaptations to their environment as constrained by genetic characteristics. As a consequence, individual trait values can be broadly explained by multiple interacting factors: macro‐level genetics in a phylogenetic context (i.e., evolutionary adaptations), micro‐level genetics (i.e., selection), trait–trait correlations, competition, and the abiotic and biotic environmental conditions at provenance (i.e., ontogeny), during growth, and at the time of measurement including phenology (Díaz et al., 2016; Garnier et al., 2017; Kattge, Díaz, et al., 2011; Kattge, Ogle, et al., 2011; Mungall et al., 2010; Violle et al., 2007). Not all of these dependencies are well studied, and their interacting effects on traits are, for most taxa, poorly characterized. For these reasons, the most useful trait data include ancillary data describing the conditions, i.e., under which the plants had grown and traits were measured. Thus, the data structure to represent trait data must include the relevant dependencies and allow for different types of ancillary data.

**FIGURE 2:**
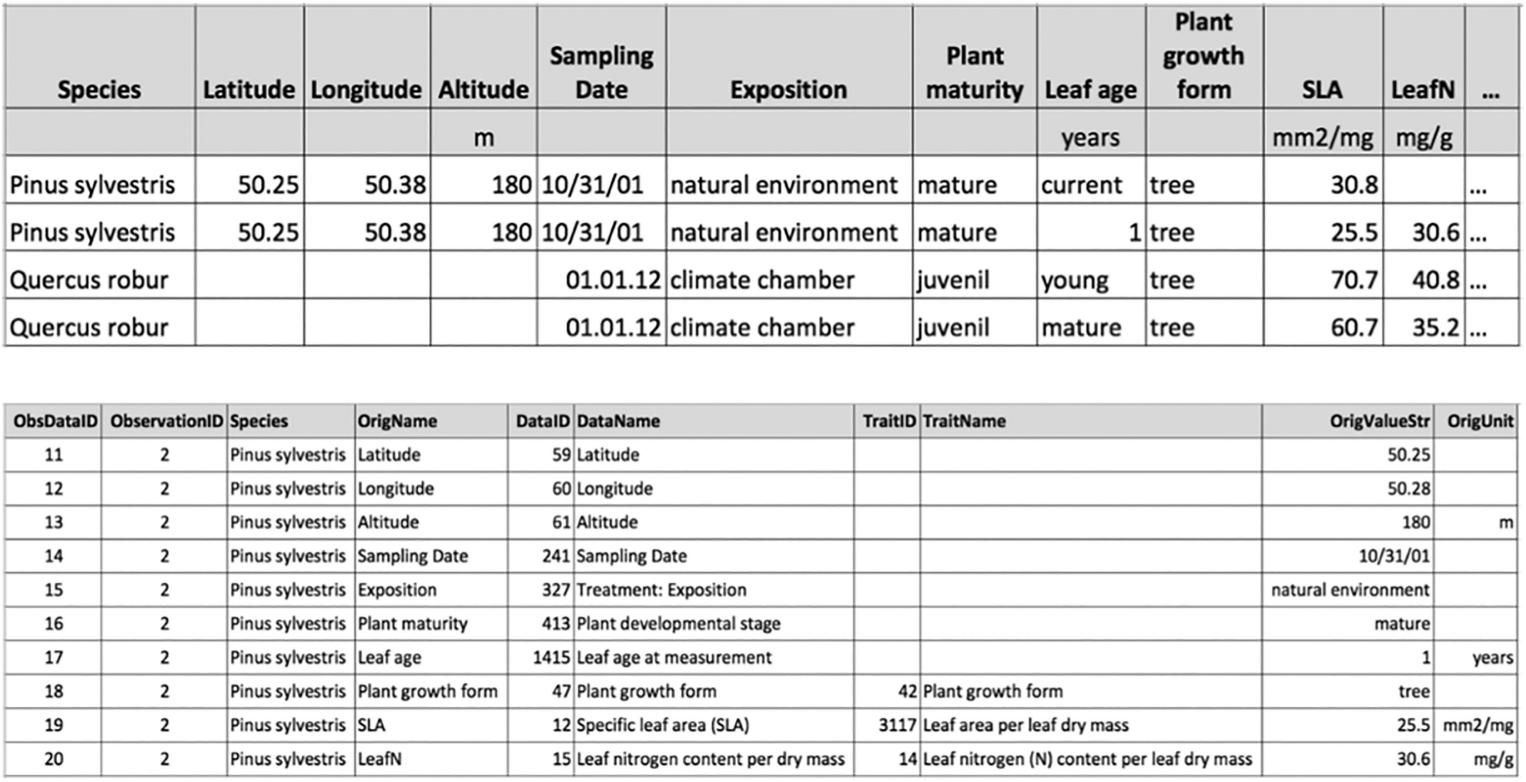
(Top) Intuitive implementation of the OBOE schema in a two‐dimensional (2D) table, with observations in rows, and traits and ancillary data in columns. (Bottom) Demonstration of the long‐table format used within TRY data releases. The second observation (row) in the top panel is provided as an example. The data release provides the unique identifiers for each data record (‘ObsDataID’), and the observation (‘ObservationID’), the taxon of the entity, and identifiers, names, values, and units of trait records and ancillary data. Empty cells for ‘TraitID's indicate that the entry is an ancillary datum. For clarity, the number of columns has been reduced compared to TRY data releases.

The structure of TRY data releases is based on the extensible observation ontology (OBOE; Madin et al., 2007) schema, implemented in a generic entity‐attribute‐value model (Kattge, Ogle, et al., 2011). The TRY database features a long‐table structure of trait records and ancillary data, with 27 columns (version 6; Table 1). Different trait records and ancillary data measured on the same entity are linked by a unique identifier (‘ObservationID’; Figure 2). The TRY data release notes (https://www.try‐db.org/TryWeb/TRY_Data_Release_Notes.pdf), distributed with each release from the TRY database, provide a more detailed overview of this data structure. Due to the size of the TRY database—15.4 million trait records and 43 million ancillary data—this can result in data releases of up to 58 million rows of trait records and ancillary data. In addition, different attributes within the released datasets are relevant for trait data filtering, i.e., trait names, species names, ancillary data, units, and identifiers for duplicates and outliers. Therefore, the process to obtain all relevant information for further analyses and discard all inconsistent data is not straightforward and there is a high risk that not all information provided for data selection is used to optimize data quality for the downstream analyses.

This paper provides an overview of the ‘rtry’ package and demonstrates its utility from a user perspective, underscoring its potential as a valuable resource for researchers grappling with the complexities of preprocessing plant trait data. By facilitating more efficient and reliable data preprocessing tasks, ‘rtry’ aims to enhance the quality of plant trait datasets for scientific inquiry.

## 2 THE ‘RTRY’ PACKAGE

To assist users in preparing the potentially huge and complex plant trait data for further analyses, the ‘rtry’ package (developed with R version 4.0) was published in 2022 by the Functional Biogeography group at the Max‐Planck‐Institute for Biogeochemistry in Jena. The stable version is available via CRAN (https://cran.r‐project.org/package=rtry) and the development version is available at the GitHub repository (https://github.com/MPI‐BGC‐Functional‐Biogeography/rtry/wiki), fostering transparency, collaboration, and continuous improvement.

Before using the ‘rtry’ package, users must install the package and load it into the R environment. The installation process automatically installs all required dependencies. Below are the commands for installing and loading the ‘rtry’ package from both CRAN and GitHub:

# install the 'rtry' package from CRAN
install.packages('rtry')

# install the 'rtry' package from GitHub
library(devtools)
devtools::install_github("MPI-BGC-Functional-Biogeography/rtry")

# load the 'rtry' package
library(rtry)

The ‘rtry’ package provides a set of functions for data preprocessing, focusing on data exploration, selection, and removal, with applicability across user levels—from beginners in R and plant trait data to experts. Leveraging the long‐table structure of data released from TRY and its accompanying features (including harmonized names for species (see data release notes; https://www.try‐db.org/TryWeb/TRY_Data_Release_Notes.pdf), harmonized names for traits and ancillary data, standardized units, and indicators for duplicates and outliers), the package is designed to empower researchers with accessible and user‐friendly functionalities that aim at streamlining a basic start‐to‐finish data preprocessing workflow. To accomplish this, ‘rtry’ adopts robust functions from the R packages ‘data.table’ (ver. 1.14.8; Barrett et al., 2024), ‘dplyr’ (ver. 1.1.2; Wickham et al., 2023), ‘tidyr’ (ver. 1.3.0; Wickham et al., 2024), and ‘utils’ (Bengtsson, 2023) in building functional commands that seamlessly align into one concise package.

By integrating a subset of existing R functions into one consistent syntax, ‘rtry’ ensures compatibility and consistency across its functions, enabling users of various skill levels to perform all necessary preprocessing procedures without the need to navigate the extensive R package ecosystem or have knowledge of various package syntaxes. For experienced R users, ‘rtry’ is complemented by comprehensive documentation, offering references for advanced preprocessing tasks. The documentation and function descriptions are part of the ‘rtry’ CRAN package, provided on the ‘rtry’ GitHub and also in the form of package vignettes which can be obtained via the R command:

# get an overview of the 'rtry' package and the corresponding documentation
help(package = 'rtry')

# name of all vignettes available
vignette(package = 'rtry')

# calling one vignette
vignette('rtry-introduction')

**FIGURE 3:**
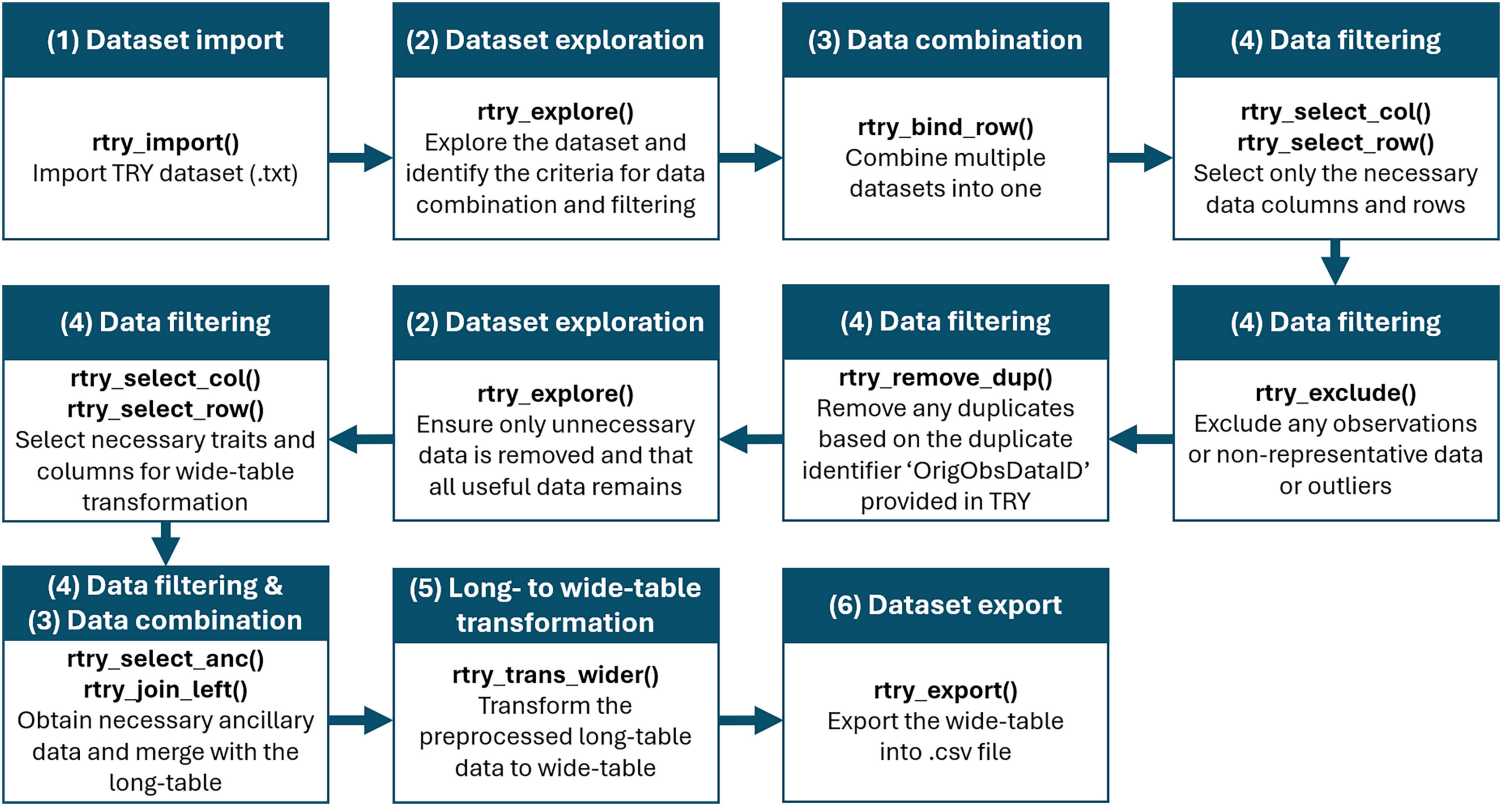
An overview of the general preprocessing workflow for TRY dataset using ‘rtry’.

To avoid potential conflicts with existing R functions, the ‘rtry’ package utilizes a naming convention where each function begins with the prefix ‘rtry_’ followed by the description of what the specific function does. Each function is designed to perform one specific data preprocessing task commonly used in plant trait data preparation. This structured approach enables users to perform a wide range of preprocessing tasks with precision and efficiency. As well, functions are kept separate to maintain feasibility for different use cases, i.e., users can use a sequence of multiple functions to suit their needs (Figures 3 and 5). The ‘rtry’ package version 1.1 consists of 16 functions (Table 2) which can be classified into six data preprocessing steps: (1) dataset import, (2) dataset exploration, (3) data combination, (4) data filtering, (5) long‐ to wide‐table transformation, and (6) dataset export, as well as the additional functionality of geocoding and reverse geocoding. Users can access the description of individual functions directly within the R environment:

# access the function description for a function, e.g., rtry_import
# including the usage and arguments of the function
?rtry_import

**TABLE 2:** List of functions inside ‘rtry’ version 1.1.

Data preprocessing step	Function	Description
Dataset import	‘rtry_import()’	Imports a text file (.txt) exported from the TRY database or comma‐separated values file (.csv)
Data exploration	‘rtry_explore()’	Groups the data based on the specified column names and provides an additional column to show the total count of each group
Data combination	‘rtry_bind_col()’	Takes a sequence of data and combines them by columns
	‘rtry_bind_row()’	Takes a sequence of data and combines them by rows
	‘rtry_join_left()’	Merges two data frames based on a specified common column and returns all records from the left data frame together with the matched records from the right data frame, all records (rows) on the right that do not exist on the left will be discarded
	‘rtry_join_outer()’	Merges two data frames based on a specified common column and returns all rows from both data, returning a joint table that contains all records (rows) from both data frames
Data filtering	‘rtry_select_col()’	Selects the specified columns from the data
	‘rtry_remove_col()’	Removes the specified columns from the data
	‘rtry_select_row()’	Selects rows based on the specified criteria and the corresponding ‘ObservationID’ from the data
	‘rtry_exclude()’	Excludes all records (rows) with the same value in the attribute specified in the argument ‘baseOn’ if the specified criteria for excluding are fulfilled for one of those records
	‘rtry_select_anc()’	Obtains a unique list of ‘ObservationID’ from the data along with the selected ancillary data (specified by ‘DataID’)
	‘rtry_remove_dup()’	Removes the duplicates from the input data using the duplicate identifier ‘OrigObsDataID’ provided within the TRY data
Long‐ to wide‐table transformation	‘rtry_trans_wider()’	Transforms the long‐table data format into a wide‐table format
Data export	‘rtry_export()’	Exports the data frame as comma‐separated values to a .csv file
Geocoding	‘rtry_geocoding()’	Uses Nominatim, a search engine for OpenStreetMap (OSM) data^a^, to perform geocoding, i.e., converting an address into coordinates (latitudes, longitudes)
	‘rtry_revgeocoding()’	Uses Nominatim, a search engine for OpenStreetMap (OSM) data^a^, to perform reverse geocoding, i.e., converting coordinates (latitudes, longitudes) into an address

^a^ The data provided by OSM are free to use for any purpose, including commercial use, and are governed by the distribution license ODbL.

Acknowledging the complexity of preprocessing plant trait data, ‘rtry’ offers an optional argument ‘showOverview’ for most functions. This optional argument provides users with a summarized dataset overview (i.e., dimension and/or column names) after each preprocessing step to enhance the usability and clarity of the ‘rtry’ package. By default, ‘showOverview’ is preset to ‘TRUE’, meaning that the dataset overview will be displayed as part of the function output, even when the users do not explicitly specify this argument. When ‘showOverview’ is set to ‘FALSE’, the overview display will be suppressed, allowing users to streamline their output and focus solely on relevant preprocessing information and tasks.

## 3 TRY DATA PREPROCESSING WORKFLOW USING ‘RTRY’

With functionalities ranging from importing and exploring the data to manipulating data using user‐defined criteria and finally exporting the preprocessed data, ‘rtry’ seamlessly facilitates data preprocessing tailored to users' specific needs across programming levels. We have outlined a general workflow for plant trait data preprocessing based on ‘rtry’ functions to assist users in understanding and applying the package's functionalities (Figure 3). The detailed workflow is available as package vignette, on CRAN, and on the GitHub Wiki. This section explains each element of this workflow and the ‘rtry’ functions involved, in the context of the generalized data preprocessing steps provided in Table 2.

### 3.1 Dataset import

The first step of the data preprocessing workflow is always the import of a dataset into the R environment. The ‘rtry_import’ function accepts five arguments—‘input’, ‘separator’, ‘encoding’, ‘quote’, and ‘showOverview’. By default, the function imports tab‐delimited text file (.txt), as exported from the TRY database. However, users have the option to modify the arguments for the separator and encoding to accommodate various file formats, such as comma‐separated values (.csv).

# import dataset released from TRY (.txt)
TRYdata <− rtry_import(<path_to_TRY_txt>)

# import dataset with comma-separated values (.csv)
# suppress the display of dimension and the column names of the imported data
data <− rtry_import(<path_to_csv>,
separator = ',',
encoding = 'UTF-8',
quote = '\"',
showOverview = FALSE)

The ‘rtry’ package contains two small datasets requested from the TRY database (‘data_TRY_15160’ and ‘data_TRY_15161’). To familiarize themselves with the data structure, users can inspect them directly in a spreadsheet‐style data viewer in RStudio and sort by ‘ObservationID’.

# open a spreadsheet-style data viewer in RStudio for sample dataset (e.g., data_TRY_15160)
View(data_TRY_15160)

With this, users can explore this dataset, for example:

- For ‘ObservationID’ 94068, there are two ‘ObsDataID’ 1021243 and 1021245, with the first one belonging to the ‘TraitID’ 3115 and the latter ancillary data. Looking deeper into the ‘DataID’ and ‘DataName’, users can see that these data “SLA: petiole excluded” are measured within “growth chambers” and could be eliminated later, depending on the research question.
- For ‘ObservationID’ 158137, users can see ancillary data with the ‘DataID’ 59, 60, 61, and 413. Looking further into the ‘ErrorRisk’ of the data “SLA: petiole excluded”, which is roughly 2.5, meaning the observation is 2.5 standard deviations away from the mean. This is probably a “good” value that users would want to keep later. As well, the ‘OrigObsDataID’ is ‘NA’, meaning that this observation is not a duplicate. Also, the “Plant developmental status” (‘DataID’ 413) could be an important information for further processing.

However, it is impossible to do so for larger datasets, which leads to the next data preprocessing step—dataset exploration.

### 3.2 Dataset exploration

The second step of the data preprocessing workflow is the exploration of the dataset. Even though the TRY data release notes (https://www.try‐db.org/TryWeb/TRY_Data_Release_Notes.pdf) provide an overview of the data structure and column headers (Table 1) of the requested dataset, they do not include the informational content of the trait records and ancillary data, which makes it challenging for preprocessing. The dataset exploration facilitated by the ‘rtry_explore’ function allows users to gain insights into the inherent traits, species, and ancillary data, enabling informed decisions and evaluation of the outcomes during preprocessing. Exploring the datasets proactively before and after each data combination or filtering step is recommended. This practice promotes data integrity and helps prevent the accidental exclusion of valuable data.

The ‘rtry_explore’ function takes four arguments—‘input’, ‘…’, ‘sortBy’, and ‘showOverview’—and organizes the input into a grouped data table based on the specified column names (‘…’). A column displaying the total count within each group is provided as additional information to the exploration. By default, the output is grouped by the first attribute when ‘sortBy’ is not specified.

The following implementation of the ‘rtry_explore’ function explores the traits and ancillary data within the imported dataset (‘TRYdata’) with the user's preferences to sort the results based on ‘TraitID’. The resulting exploration output (‘TRYdata_explore’) presents all traits followed by the ancillary data (identified by the missing value—‘NA’—in ‘TraitName’ and ‘TraitID’). The main purpose of this exploration is to obtain an overview of traits, and of ancillary data and sub‐traits (indicated by different ‘DataID’ under the same ‘TraitID’) available for data filtering.

# group the input data (TRYdata) based on DataID, DataName, TraitID, and TraitName
# and sort the output by TraitID using the sortBy argument
# not show dimension and the column names of the exploration result
TRYdata_explore <− rtry_explore(TRYdata,
 DataID, DataName, TraitID, TraitName,
 sortBy = TraitID,
 showOverview = FALSE)

Data exploration can also be used to obtain the species information for which data are available by including the column headers ‘AccSpeciesID’ and/or ‘AccSpeciesName’ within the argument ‘…’. However, users should be aware that an exploration on species, traits, and sub‐traits simultaneously may result in a long list of results due to the potentially diverse dataset.

### 3.3 Data combination

**FIGURE 4:**
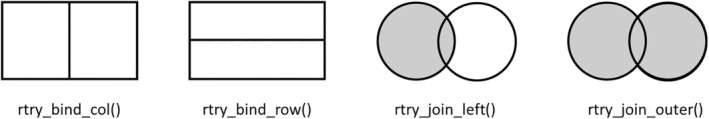
Visual interpretation of the four data combination functions provided by ‘rtry’.

Given the diverse origins of plant trait data, users frequently encounter the need to manage multiple datasets during preprocessing. To facilitate this, ‘rtry’ provides four data combination functions, namely ‘rtry_bind_col()’, ‘rtry_bind_row()’, ‘rtry_join_left()’, and ‘rtry_join_outer()’. The visual interpretation of these functions is shown in Figure 4.

The ‘rtry_bind_col’ and ‘rtry_bind_row’ functions take a list of data frames (‘…’), enabling users to combine data frames either by columns or by rows. Since these two functions do not consider a common attribute, users must ensure the proper ordering of columns, respectively, rows, before binding.

# combine multiple TRY datasets (TRYdata1, TRYdata2, TRYdata3) already imported into R by row
TRYdata_combine <− rtry_bind_row(TRYdata1, TRYdata2, TRYdata3)

In contrast, the ‘rtry_join_left’ and ‘rtry_join_outer’ functions merge two data frames (‘x’ and ‘y’) based on a common attribute (‘baseOn’). The ‘rtry_join_left’ function returns the left data (‘x’) with the matched records from the right data frame (‘y’), while the ‘rtry_join_outer’ function returns all records from both data frames (‘x’ and ‘y’).

# merge the georeferenced information (georef) to the dataset (TRYdata)
# based on the common identifier ObservationID
# output all records in TRYdata with additional columns containing the georeferenced information
# suppress overview display
TRYdata_georef <− rtry_join_left(TRYdata, georef, baseOn = ObservationID, showOverview = FALSE)

# merge two datasets containing coordinates (coord) and locations (loc)
# based on the common identifier ObservationID
# it does not matter if certain ObservationID occurs only in one dataset
georef <− rtry_join_outer(coor, loc, baseOn = ObservationID)

### 3.4 Data filtering

A major goal of data preprocessing is data filtering. This functionality is especially crucial for datasets retrieved from the TRY database, as they often contain more information than necessary for user objectives and trait data inconsistent with planned analyses. To avoid incorporating substantial data filtering in their downstream analyses—which is possible but prone to errors and reduces computational efficiency—it is essential to extract (select) relevant information or remove (exclude) irrelevant information beforehand. ‘rtry’ offers six functions to facilitate this data filtering process: ‘rtry_select_col()’, ‘rtry_remove_col()’, ‘rtry_select_row()’, ‘rtry_exclude()’, ‘rtry_select_anc()’, and ‘rtry_remove_dup()’.

#### 3.4.1 Filtering attributes (columns) from the dataset

In TRY version 6, the output table has 27 columns (Table 1), encompassing trait or ancillary data measurements and informational content recognizing the data contributors and contributed datasets. To select only the relevant columns from the imported datasets, users can employ either the ‘rtry_select_col’ or ‘rtry_remove_col’ function. These two functions accept three arguments—an imported data frame (‘input’), a list of column names to be selected or removed (‘…’), and ‘showOverview’. While ‘rtry_select_col()’ allows users to explicitly select a list of columns to retain, ‘rtry_remove_col()’ removes the specified columns. In general, it is more convenient to use the ‘rtry_remove_col’ function for removing only a small fraction of the data frame. It is important to note that the column containing unique identifiers for each observation (‘ObservationID’) and for duplicate trait records (‘OrigObsDataID’) from the TRY dataset should not be removed to ensure the proper functionality of the later preprocessing steps, such as data selection and duplicate removal.

# remove six columns from the imported data (TRYdata)
TRYdata_simplified <- rtry_remove_col(TRYdata,
LastName, FirstName, DatasetID, Dataset, Reference, Comment)

#### 3.4.2 Filtering records (rows) from the dataset

The ‘rtry_select_row’ and ‘rtry_exclude’ functions allow users to select or exclude records (rows) for further analyses based on their relevance or consistency. While the TRY database provides the trait names and corresponding identifiers on the data explorer (https://www.try‐db.org/de/de.php), it does not offer a comprehensive list of the sub‐traits or the ancillary data. Therefore, conducting data exploration using ‘rtry_explore()’ (Section 3.2) is essential beforehand to obtain the informational content, such as the traits, sub‐traits, and ancillary data available within the datasets.

The ‘rtry_select_row’ function accepts five arguments—a data frame (‘input’), criteria for selection (‘…’), and three optional arguments ‘getAncillary’, ‘rmDuplicates’, and ‘showOverview’. This function keeps the rows that fulfill the specified criteria (‘…’) from the data frame (‘input’). Users can keep all ancillary data that share the same unique identifiers for each observation in TRY (‘ObservationID’) of the retained rows by setting the argument ‘getAncillary’ to ‘TRUE’. Additionally, users have the option to remove duplicates from the datasets by setting ‘rmDuplicates’ to ‘TRUE’, invoking the ‘rtry_remove_dup’ function, which will be introduced later in this section.

Among all functions within ‘rtry’, ‘rtry_exclude()’ is considered to be the most valuable when preprocessing plant trait data because it provides flexible arguments to filter trait measurements and respective ancillary data. The ‘rtry_exclude’ function accepts four arguments—a data frame (‘input’), criteria for exclusion (‘…’), the attribute on which exclusion is based (‘baseOn’), and the optional argument ‘showOverview’. This function removes data from the data frame (‘input’) based on the specified criteria (‘…’). Users are required to explicitly set the argument ‘baseOn’ to an identifier that they see fit. For example, when set to ‘ObservationID’, ‘rtry_exclude()’ removes all records of the respective entities (indicated by the same ‘ObservationID’) from a data frame if the specified criterion for exclusion is fulfilled for any record. Accordingly, if ‘baseOn’ is set to the unique identifier of the consolidated species name (‘AccSpeciesID’), all records of the corresponding species will be excluded if the criterion is met for any one record of that species. Alternatively, when ‘baseOn’ is set to ‘ObsDataID’, the unique identifier for each record or row in the TRY dataset, the function will exclude only the individual records for which the specified criterion is fulfilled.

Below are three examples of data selection and exclusion. Detailed explanations and implementations can be found in the package vignettes, on CRAN, and the GitHub Wiki.

##### Example 1: Select relevant trait records and ancillary data

This example selects only data from the complex plant trait dataset considered relevant for further analyses. Users can explore the dataset first to obtain an overview of the available traits and ancillary data within the dataset, then identify the criteria for selecting the relevant trait records and ancillary data for further preprocessing and analyses.

# explore the traits (TraitID > 0) and ancillary data (TraitID == NA) inside the dataset (TRYdata)
TRYdata_explore <- rtry_explore(TRYdata,
 DataID, DataName, TraitID, TraitName,
 sortBy = TraitID)

# select trait records related to leaf area per leaf dry mass, i.e., TraitIDs 3115, 3116, 3117
# and simultaneously select relevant ancillary data (specified by DataID):
# 59 Latitude; 60 Longitude; 61 Altitude; 6601 Sampling date; 327 Exposition
# 413 Plant developmental status / plant age / maturity / plant life stage
# 1961 Health status of plants (vitality); 113 Reference / source
TRYdata_select <- rtry_select_row(TRYdata,
TraitID %in% c(3115, 3116, 3117) | DataID %in% c(59, 60, 61, 6601, 327, 413, 1961, 113))

##### Example 2: Remove all observations on non‐mature plants

This example removes all non‐mature plant observations while keeping those measured from the mature plants. Through the dataset exploration in Example 1, users learn that ‘DataID’ 413 provides information on plant developmental status or maturity. Here, the ‘DataID’ 413 is used to perform another dataset exploration, and the obtained values (‘OrigValueStr’) for plant maturity are used to identify criteria for filtering. While ‘rtry_exclude()’ removes all records of the whole observation measured from a non‐mature plant, it is worth noting that this example also keeps the observations where the developmental state is explicitly unknown or is not provided (no ‘DataID’ 413 for the given observation), with the assumption that the measurements followed the recommended measurement protocol—measuring traits on mature plants.

# subset of dataset (TRYdata) with only the rows containing plant developmental status (DataID 413)
TRYdata_subset <- rtry_select_row(TRYdata, DataID %in% 413)

# explore the different plant development states within the data subset (TRYdata_subset)
# sort the exploration by OrigValueStr to obtain the developmental states in alphabetical order
# note: no StdValue available for DataID 413, since developmental status is not a continuous trait
TRYdata_subset <- rtry_explore(TRYdata_subset,
 DataID, DataName, OrigValueStr, OrigUnitStr,
 sortBy = OrigValueStr)

# remove all observations (ObservationID) that are measured on non-mature plants
# criteria:
# 1. DataID equals 413 - Plant developmental status / plant age / maturity / plant life stage
# 2. OrigValueStr equals "juvenile" or "saplings" (identified in dataset exploration)
TRYdata_exclude <- rtry_exclude(TRYdata,
 (DataID %in% 413) & (OrigValueStr %in% c("juvenile", "saplings")),
 baseOn = ObservationID)

##### Example 3: Remove outliers

To remove the outliers identified during data integration of the TRY database, users can take advantage of the column ‘ErrorRisk’ provided inside the data released from the database. The ‘ErrorRisk’ quantifies the maximum distance of the trait record from a respective mean at the species, genus, or family level in terms of standard deviation (a modified z‐transformation; Kattge, Díaz, et al., 2011; Kattge et al., 2020). After exploring the dataset for potential outliers, this example filters the data with ‘ErrorRisk’ larger than or equal to 3.0. Note that this time the argument ‘baseOn’ is set to ‘ObsDataID’, as we intend to exclude only the outliers for individual trait records while keeping the rest of the observation which might have other relevant trait measurements or ancillary information.

# explore the input data (TRYdata) based on DataID, DataName, TraitID, TraitName, and ErrorRisk
# sort the output by ErrorRisk
TRYdata_explore <- rtry_explore(TRYdata,
 DataID, DataName, TraitID, TraitName, ErrorRisk,
 sortBy = ErrorRisk)

# remove outliers: individual trait records (ObsDataID) identified with ErrorRisk >= 3
# while keeping the rest of the observations in the dataset (TRYdata)
TRYdata_exclude <- rtry_exclude(TRYdata,
 ErrorRisk >= 3,
 baseOn = ObsDataID)

#### 3.4.3 Removing duplicates

As of October 2022, the TRY database comprised 696 datasets from 1108 data contributors (Boenisch & Kattge, 2023). To keep track of potential duplicate entries, a unique identifier ‘OrigObsDataID’ was assigned when there was a high probability that the same trait records had previously been contributed to TRY. This determination is based on the criteria: (1) same ‘TraitName’, ‘AccSpeciesName’, and ‘UnitName’, (2) similar ‘StdValue’—accounting for rounding effects, and (3) not different geographic coordinates, which were assessed using standardized latitude and longitude (Kattge et al., 2020).

Within ‘rtry’, we provide the ‘rtry_remove_dup’ function for users to easily remove the duplicates within a data frame (‘input’) based on the identifier ‘OrigObsDataID’. While the dimension of the resulting data frame can be suppressed by setting ‘showOverview’ to ‘FALSE’, the number of duplicates removed will still be shown. Users should be aware that if the original, not duplicate, trait record was not requested from TRY (e.g., if only public data or specific datasets were requested from TRY and the original trait record was part of the restricted data or another dataset), the duplicates identified by TRY will still be removed by this function, resulting in data loss.

# remove the duplicates within the dataset (TRYdata)
TRYdata_rm_dup <− rtry_remove_dup(TRYdata)

### 3.5 Long‐table to wide‐table transformation

Trait datasets can be structured in either long‐ or wide‐table formats. The data released from TRY are given in a long‐table format, which allows a consistent structure as different traits or ancillary data are stored in separated rows (i.e., simply add or remove rows when needed, instead of having empty columns for missing information). The long‐table format keeps this type of data in a denser format and is more flexible for data storage. Yet, a wide‐table format is often more convenient for analyses as a tabular view is more straightforward to visually interpret and assess. Therefore, the ‘rtry’ package provides the ‘rtry_trans_wider’ function to transform the preprocessed trait data from long‐ to wide‐table format for further analyses. This function accepts five arguments—a data frame (‘input’), the columns from which the output column names and values are to be obtained (‘names_from’ and ‘values_from’), the optional argument to define the function applied to the output values when necessary (‘values_fn’), and whether to display the dimension of the resulting wide‐table (‘showOverview’).

Several preprocessing steps are necessary before performing the long‐ to wide‐table transformation on the TRY dataset. The first step is to select only traits with numerical values and relevant columns (else the attribute in ‘values_fn’ might cause error). Next, users can obtain a list of relevant ancillary data from the original dataset as needed, e.g., georeferencing information like latitude and longitude indicated by ‘DataID's 59 and 60, respectively. The ‘rtry’ package provides the ‘rtry_select_anc’ function to facilitate this step. The ‘rtry_select_anc’ function takes three arguments—an imported data frame (‘input’), a list of ‘DataID's of the ancillary data to be selected (‘…’), and the optional argument ‘showOverview’. This function returns a unique list of ‘ObservationID’ and the corresponding ancillary data of interest. When the ancillary data (latitude and longitude in this case) are extracted, they can be merged to the numerical traits using 'rtry_join_left()' to include the ancillary data in the resulting wide‐table.

Once the data are prepared, transformation can be performed using the ‘rtry_trans_wider’ function, as demonstrated below. To ensure successful transformation when dealing with the potential existence of multiple records for a single trait under one ‘ObservationID’ (e.g., multiple measurements of specific leaf area of one observation entity), we recommend defining the argument ‘values_fn’ either by mean (‘mean’) or, if more appropriate, by maximum (‘max’) or minimum (‘min’). If this argument is not specified, trait records (same ‘TraitID’) with different ‘DataID's under the same ‘OberservationID’ will be displayed within the same cell as text, causing errors in numerical data analyses.

# provide the standardized trait values per observation, together with species names
# and the georeferences of the sampling site (59: Latitude and 60: Longitude), if available,
# in a wide table format; several steps are necessary:

# 1. select only the trait records that have standardized numeric values from the dataset (TRYdata)
# the complete.cases() is used to ensure the cases are complete, i.e. have no missing values
num_traits <- rtry_select_row(TRYdata,
complete.cases(TraitID) & complete.cases(StdValue))

# 2. select the relevant columns for transformation, while suppress the data overview display
num_traits <- rtry_select_col(num_traits,
ObservationID, AccSpeciesID, AccSpeciesName, TraitID, TraitName,
StdValue, UnitName,
showOverview = FALSE)

# 3. extract latitude (DataID 59) and longitude (DataID 60) of each observation within TRYdata
# while suppress the data overview display
georef <- rtry_select_anc(TRYdata,
 59, 60,
 showOverview = FALSE)

# 4. merge the relevant data frames based on the ObservationID using rtry_join_left()
num_traits_georef <- rtry_join_left(num_traits, georef, baseOn = ObservationID)

# 5. perform wide table transformation of TraitID, TraitName, and UnitName based on
# ObservationID, AccSpeciesID, and AccSpeciesName with cell values from StdValue
# if several records with StdValue were provided for one trait with the same
# ObservationID, AccSpeciesID, and AccSpeciesName, calculate their mean
num_traits_georef_wider <- rtry_trans_wider(num_traits_georef,
 names_from = c(TraitID, TraitName, UnitName),
 values_from = c(StdValue),
 values_fn = list(StdValue = mean))

### 3.6 Dataset export

The ‘rtry_export’ function can be used to save the preprocessed data in their final structure (either in long‐ or wide‐table format) as comma‐separated‐values into a .csv file at a specified directory. This function takes four arguments—the data to be saved (‘data’), the output path (‘output’), and two optional arguments that by default insert double quotes around any character or factor columns (‘quote’), and sets the file to “UTF‐8” encoding (‘encoding’).

# export the preprocessed data (TRYdata) to a specific directory (e.g., in the temporary directory)
rtry_export(TRYdata, file.path(tempdir(), "TRYdata_preprocessed.csv"))

## 4 ADDITIONAL USE CASES USING ‘RTRY’

While the TRY database serves as a central resource for plant trait data, researchers often draw from diverse sources to enrich their analyses. Building upon the foundational functionality of ‘rtry’ in plant trait data preprocessing, we have provided additional example workflows that encompass the geocoding and reverse geocoding procedures and the application of ‘rtry’ to data acquired from sources other than the TRY database. The detailed example workflow for (reverse) geocoding can be found as a package vignette on CRAN, whereas the ‘rtry’ GitHub Wiki provides the vignettes for geocoding and the preprocessing workflow for the NEON plant trait data.

### 4.1 Geocoding and reverse geocoding

Georeferencing is necessary to assess the plausibility of location information, filter data using a common coordinate system, estimate geographic patterns, link to georeferenced—e.g., environmental—data, and address the spatial autocorrelation of the plant trait data.

There are two functions within ‘rtry’ to assist users with geocoding (‘rtry_geocoding()’ derives latitude and longitude for a given location name) and reverse geocoding (‘rtry_revgeocoding()’ derives the location name from provided latitude and longitude) for a list of locations or coordinates in the WGS84 Coordinate System. These functions rely on Nominatim, a search engine for OpenStreetMap (OSM) data. The data provided by the OSM are freely available for any purpose, including commercial use, and are governed by the Open Database License (ODbL; https://wiki.osmfoundation.org/wiki/Licence). Users should note that an absolute maximum of one request per second (no heavy usage) and a valid email address to identify the request are required when using the OSM service as part of the Nominatim Usage Policy (details can be found on: https://operations.osmfoundation.org/policies/nominatim/).

While the example workflow provides the script for obtaining the coordinates or locations from a list of corresponding information, these two functions can also be applied to individual entries—‘rtry_geocoding()’ requires a string of an address (‘address’) and ‘rtry_revgeocoding()’ requires a data frame containing latitude and longitude (‘lat_lon’).

# convert the address of MPI-BGC ("Hans-Knoell-Strasse 10, 07745 Jena, Germany")
# into coordinates in latitudes and longitudes
# note: please change to your own email address when executing this function
rtry_geocoding("Hans-Knoell-Strasse 10, 07745 Jena, Germany",
 email = "john.doe@example.com")

# convert the coordinates (must be a data frame) of MPI-BGC (50.9101, 11.56674) into an address
# note: please change to your own email address when executing this function
rtry_revgeocoding(data.frame(50.9101, 11.56674),
 email = "john.doe@example.com")

### 4.2 Preprocessing NEON plant foliar trait data

The National Ecological Observatory Network (NEON) program is a research platform funded by the United States National Science Foundation (NSF) that provides free and long‐term data across biomes comprising the continental U.S. and Hawaii on key ecological metrics as a basis to discover and understand the impacts of climate change (NEON, 2023). We have chosen the plant foliar traits dataset (product ID: DP1.10026.001) from the NEON data portal (NEON, 2016) to demonstrate a use case of the ‘rtry’ package outside of plant trait data from TRY. The NEON plant foliar traits dataset contains trait measurements (leaf mass per area, leaf water content, chlorophyll, carbon and nitrogen concentrations and stable isotopes, major and minor elements, and lignin) of sun‐lit canopy foliage at either individual (woody plants) or community (herbaceous plants) levels (NEON, 2016).

**FIGURE 5:**
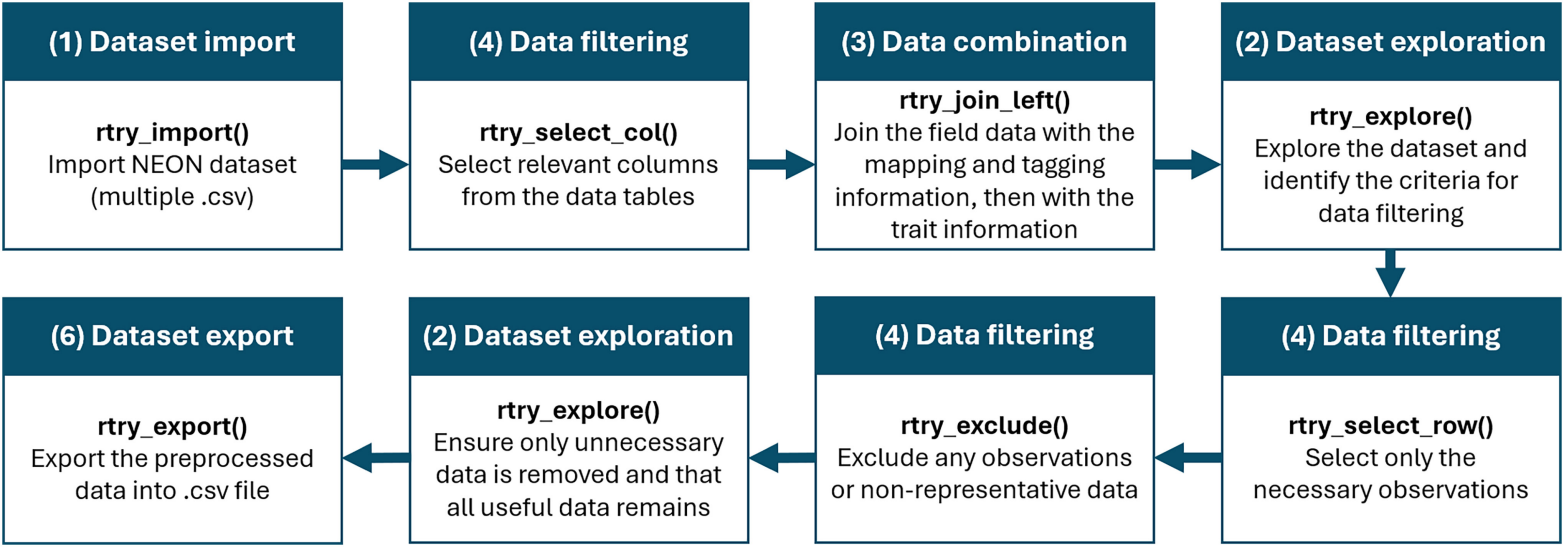
An overview of the general preprocessing workflow for NEON dataset using ‘rtry’.

While the detailed example is available on the GitHub Wiki, this section provides an overview of the preprocessing steps using ‘rtry’ for NEON data (Figure 5). The objective is to demonstrate the versatility of the ‘rtry’ package beyond the TRY database and illustrate how users can seamlessly chain together various functions within the package to suit the needs of cross‐cutting and integrative analyses.

#### 4.2.1 Dataset import

Upon downloading the NEON dataset, users receive multiple .csv files, each representing a different data table. Information about each data table can be found in the user guide (https://data.neonscience.org/data‐products/DP1.10026.001#documentation). Users can employ the ‘rtry_import’ function to import the NEON dataset into the R workspace.

# for the list of NEON data within the NEON_output/stackedFiles directory
# read the .csv files and assign them to a corresponding variable
for (i in list.files(path = paste0(NEON_output, "/stackedFiles") -> ipath,
pattern = "vst|cfc")){
assign(file_path_sans_ext(i),
rtry_import(paste0(ipath, "/", i),
separator = ",",
encoding = "UTF-8",
quote = "\"",
showOverview = FALSE))
}

#### 4.2.2 Data filtering and combination

Similar to the TRY data, the NEON plant trait data also contain more information than necessary for data preprocessing. For demonstration purposes, the script below utilizes the ‘rtry_select_col’ function to obtain the data columns relevant to the field collection of foliar samples (‘cfc_fieldData’), the location information of individual stems (‘vst_mappingandtagging’), and the leaf mass per area (LMA) measurement of the foliar samples (‘cfc_LMA’).

# select the necessary columns from the corresponding data table
# 1. field data
fieldData <− rtry_select_col(cfc_fieldData,
individualID, sampleID, namedLocation, domainID, siteID, plotID,
subplotID, geodeticDatum, decimalLatitude, decimalLongitude,
elevation, sampleType, taxonID, scientificName, plantStatus)

# 2. geolocation information for finding the stem locations of woody sampled plants
mappingAndTagging <− rtry_select_col(vst_mappingandtagging,
individualID, pointID, stemDistance, stemAzimuth)

# 3. leaf mass per area (LMA) of foliar samples
lma <- rtry_select_col(cfc_LMA,
sampleID, lmaSampleID, lmaSampleCode, dryMass, scannedLeafNumber,
leafArea, leafMassPerArea, dryMassFraction)

Next, the ‘rtry_join_left’ function is used to merge the mapping and tagging information and the trait information (e.g., LMA) with the field data, using the unique identifiers ‘individualID’ and ‘sampleID’ within the NEON data tables. Information regarding which identifier to use for merging can also be found in the user guide provided by NEON. It is important to note that duplicates may be present within the ‘vst_mappingandtagging’ table due to certain individuals being remapped occasionally to address geolocation issues. To address this, the ‘unique’ function is introduced to the mapping and tagging information during merging.

# join field data with the mapping and tagging information based on the identifier (individualID)
fullTable <− rtry_join_left(fieldData, unique(mappingAndTagging), baseOn = individualID)

# join the merged data with the trait records, i.e., lma, sample on the sampleID
fullTable <− rtry_join_left(fullTable, lma, baseOn = sampleID)

#### 4.2.3 Dataset exploration and data filtering

At this point, users have obtained a single table that contains all traits and georeferenced information necessary to proceed. Users can then use the ‘rtry_explore’ function to review the dataset to identify criteria for excluding observations, non‐representative data, or outliers. The identified criteria can be used to select and exclude any observations accordingly using the ‘rtry_select_row’ or ‘rtry_exclude’ functions. It is highly recommended to use the ‘rtry_explore’ function again after each filtering step to verify that only the intended unnecessary data were removed and that all desired useful data remain. This iterative approach to data exploration and filtering ensures the integrity of the dataset and the reliability of subsequent analyses. Here, we provide two examples for this preprocessing task.

##### Example 1: Filtering data with geolocation information

The first example is to obtain data that have geolocation information, indicated with the identifier for a point location (‘pointID’), the horizontal distance from stem to the ‘pointID’ location (‘stemDistance’), and the azimuth relative to True North between stem and ‘pointID’ location (‘stemAzimuth’). Within the NEON data, each record has a plot‐level location which may be sufficient for some applications. For more precise locations of individual stems, precise coordinates must be calculated using the mapping and tagging information. To do so, users can begin by assessing how many records lack the required mapping and tagging information using the ‘rtry_explore’ function. The column ‘siteID’ is also used for a better understanding of the datasets during this data exploration, in addition to the three geolocation location columns that are required for calculating the precise location of an individual stem. Once the existence of missing geolocation information is confirmed, users can either use ‘rtry_select_row()’ to select only the data with geolocation information, or they can use ‘rtry_exclude()’ to exclude the data without geolocation information. Afterward, data exploration is used to verify the datasets—ensure all necessary information is retained and all unnecessary information is removed.

# explore the location information in the full table to identify filtering criteria
df_explore_before <− rtry_explore(fullTable,
siteID, pointID, stemDistance, stemAzimuth,
sortBy = pointID)

# method 1: selecting only the data with geolocation information
# criteria: none of the three geolocation columns has "NA" value
fullTable_geoloc <− rtry_select_row(fullTable,
(!is.na(pointID) & !is.na(stemDistance) & !is.na(stemAzimuth)))

# method 2: excluding the data without geolocation information
# criteria: either one of the three geolocation columns has "NA" value
fullTable_geoloc <− rtry_exclude(fullTable,
(is.na(pointID) | is.na(stemDistance) | is.na(stemAzimuth)),
baseOn = sampleID)

# explore the location information in the full table again
df_explore_after <- rtry_explore(fullTable_geoloc,
siteID, pointID, stemDistance, stemAzimuth,
sortBy = pointID)

##### Example 2: Filtering data from healthy individuals

The second example involves filtering the dataset to obtain only healthy individuals based on the ‘plantStatus’ column within the NEON plant trait dataset. Again, data exploration with ‘rtry_explore()’ is essential to identify the criteria for data filtering. This time, exploration focuses on the columns ‘siteID’, ‘plotID’, ‘subplotID’, ‘scientificName’, and ‘plantStatus’, allowing users to gain insights into the different plant physical statuses, and the physical status distribution among sites and species. Sorting the exploration results by scientific names enhances clarity. By inspecting the exploration result, users have an overview of the different plant physical statuses (e.g., “OK”, “Disease damaged”, and “Insect damaged”) associated with each species within the datasets. These serve as keywords for filtering healthy plant records through the ‘rtry_select_row’ and ‘rtry_exclude’ functions. Another data exploration is recommended after data filtering to ensure all the damaged individuals were successfully removed, and only healthy ones are retained in the dataset.

# explore the relevant columns in the full table to identify filtering criteria
df_explore_before <− rtry_explore(fullTable_geoloc,
siteID, plotID, subplotID, scientificName, plantStatus,
sortBy = scientificName)

# method 1: selecting only the healthy individuals
# criteria: plantStatus equals to OK
fullTable_geoloc_healthy <− rtry_select_row(fullTable_geoloc,
(plantStatus == "OK"))

# method 2: excluding the damaged individuals
# criteria: plantStatus equals either Disease damaged or Insect damaged
fullTable_geoloc_healthy <− rtry_exclude(fullTable_geoloc,
(plantStatus %in% c("Disease damaged", "Insect damaged")),
baseOn = sampleID)

# explore the relevant columns in the full table
df_explore_after <− rtry_explore(fullTable_geoloc_healthy,
siteID, plotID, subplotID, scientificName, plantStatus,
sortBy = scientificName)

#### 4.2.4 Dataset export

Once the data preprocessing is completed, the ‘rtry_export’ function can be used to export the preprocessed NEON trait data into comma‐separated values (.csv) file.

# export the preprocessed NEON data into a .csv file
output_file <− file.path(NEON_output, paste0(basename(NEON_output), ".csv"))
rtry_export(fullTable_geoloc_healthy, output_file)

## 5 CONCLUSION

This paper introduces the open‐source R package ‘rtry’ from a user perspective. By offering a curated selection of functions essential to data preprocessing tasks, ‘rtry’ empowers users of all skill levels in R and plant traits to efficiently explore, filter, and reformat trait records based on their needs without delving into the complex ecosystem of R packages. The accessible and comprehensive package documentation and example workflows on various platforms ensure that even users unfamiliar with R or the inherent data structure of trait data can easily navigate and utilize its functionalities to streamline the preprocessing workflow of plant trait data.

We demonstrate the versatility of ‘rtry’ extends beyond the TRY database, showcasing its applicability in preprocessing plant trait datasets acquired from other platforms such as the NEON program. This illustrates the adaptability and utility of ‘rtry’ across diverse datasets, reinforcing its role in ecological research and data analysis.

In conclusion, ‘rtry’ offers researchers a robust and user‐friendly solution within a single package for preprocessing plant trait data. Its accessibility, functionality, and versatility make it a useful tool for researchers aiming to harness the potential of their plant trait datasets.

## AUTHOR CONTRIBUTIONS

**Olee Hoi Ying Lam:** Conceptualization (equal); data curation (equal); methodology (equal); project administration (supporting); software (lead); validation (lead); visualization (lead); writing – original draft (lead); writing – review and editing (lead). **Jens Kattge:** Conceptualization (equal); data curation (equal); funding acquisition (equal); methodology (equal); project administration (lead); resources (equal); software (supporting); supervision (lead); validation (supporting); visualization (supporting); writing – original draft (supporting); writing – review and editing (equal). **Susanne Tautenhahn:** Methodology (supporting); software (supporting); validation (supporting); writing – review and editing (equal). **Gerhard Boenisch:** Data curation (equal); methodology (supporting); resources (equal); validation (supporting); writing – review and editing (equal). **Kyle R. Kovach:** Methodology (supporting); software (supporting); validation (supporting); writing – review and editing (equal). **Philip A. Townsend:** Funding acquisition (equal); writing – review and editing (equal).

## FUNDING INFORMATION

P.A.T. and K.R.K. acknowledge funding support from NSF Macrosystems Biology and NEON‐Enabled Science (MSB‐NES) award DEB 1638720 and NSF ASCEND Biology Integration Institute (BII) award DBI 2021898. Additional support for O.H.Y.L. and P.A.T. was provided by UW‐Madison USDA Hatch award WIS03079 and NASA AIST grant 80NSSC20K0208. O.H.Y.L. was funded by the Max Planck Institute for Biogeochemistry (MPI‐BGC) for part of the development process.

## CONFLICT OF INTEREST STATEMENT

The authors declare no conflict of interest.

## DATA LICENSE

The ‘rtry’ package is distributed under the CC BY 4.0 license (https://creativecommons.org/licenses/by‐nc‐nd/4.0/), with a remark that the (reverse) geocoding functions provided within the package used the Nominatim developed with OpenStreetMap (OSM). Although the OSM API and the data provided are free to use for any purpose, including commercial use, they are governed by the Open Database License (ODbL; https://wiki.osmfoundation.org/wiki/Licence).

## Data Availability

The R package ‘rtry’ is available from CRAN (https://cran.r‐project.org/package=rtry) and the development version can be accessed at the GitHub repository (https://github.com/MPI‐BGC‐Functional‐Biogeography/rtry). Comprehensive package documentation and vignettes describing detailed data preprocessing workflows can be accessed from CRAN (https://cran.r‐project.org/package=rtry) and the GitHub Wiki (https://github.com/MPI‐BGC‐Functional‐Biogeography/rtry/wiki). The data in example workflows are provided within the ‘rtry’ package and the NEON data portal (https://data.neonscience.org/data‐products/DP1.10026.001).
